# General lighting can overcome accidental viewing

**DOI:** 10.1177/20416695231215604

**Published:** 2023-12-06

**Authors:** Isabelle Bülthoff, Martin Breidt, Heinrich H. Bülthoff, Daniel Kersten

**Affiliations:** 28328 Max Planck Institute for Biological Cybernetics, Tübingen, Germany; 28328 Max Planck Institute for Biological Cybernetics, Tübingen, Germany; 28328 Max Planck Institute for Biological Cybernetics, Tübingen, Germany; Psychology Department, University of Minnesota, Minneapolis, USA

**Keywords:** illumination, shadow, accidental viewpoint, perception, object recognition, scene recognition

## Abstract

When seeing an object in a scene, the presumption of seeing that object from a general viewpoint (as opposed to an accidental viewpoint) is a useful heuristic to decide which of many interpretations of this object is correct. Similar heuristic assumptions on illumination quality might also be used for scene interpretation. Here we tested that assumption and asked if illumination information helps determine object properties when seen from an accidental viewpoint. Test objects were placed on a flat surface and illumination was varied while keeping the objects’ images constant. Observers judged the shape or rigidity of static or moving simple objects presented in accidental view. They also chose which of two seemingly very similar faces was familiar. We found: (1) Objects might appear flat without shadow information but were perceived to be volumetric objects or non-planar in the presence of cast shadows. (2) Apparently non-rigid objects became rigid with shadow information. (3) Shading and shadows helped to infer which of two face was the familiar one. Previous results had shown that cast shadows help determine spatial layout of objects. Our study shows that other properties of objects like rigidity or 3D-shape can be disambiguated by shadow information.

Shadows are a very special kind of visual stimuli (for review, see Casati, 2004; Mamassian et al., 1998; Santos et al., 2018), as they can hinder or help scene interpretation. Computational studies have underscored the difficulty of reliably extracting useful information from cast shadows for object identification, shape, and relative depth. In computer vision, cast shadows have been largely treated as nuisance factors to be filtered out (Dee & Santos 2011; Li & Li, 2022; López-Torres et al., 2020). One of the reasons for this is the complex and highly variable nature of illumination, making it difficult to tease apart shadow regions from objects. The computational challenge is further exacerbated by the shadow correspondence problem—reliably determining which shadows (and features) belong to which of the casting surfaces (Mamassian, 2004). So, while the recovery of shape and depth from motion and stereo is much better understood, the conditions under which cast shadows are useful for estimating object properties such as shape, rigidity, and relative depth is less clear.

Behavioral studies are also consistent with the problematic nature of cast shadows. For example, cast shadows can hamper correct or speed interpretation by reducing the visibility of other visual information, or be dismissed early in image processing (Braje et al., 1998; Ehinger et al., 2016; Khuu et al., 2014; Rensink & Cavanagh, 2004). Observers often fail to detect shadow inconsistencies (Mamassian, 2004; Nightingale et al., 2019; Ostrovsky et al., 2005). On the positive side, cast shadows can help by providing additional information for recognizing objects (Castiello, 2001), for example because a cast shadow always reveals an unseen profile of the object (Casati, 2004). Behavioral studies have also shown conditions under which cast shadows can be used in the estimation of the spatial relationships between objects (Mamassian et al., 1998) or in the recognition of objects (Bonfiglioli et al., 2004). Additionally, how well observers can use them for inferring light direction and what priors might play a role in those inferences have been examined (e.g., Mamassian & Goutcher, 2001). Given the complex nature of shadows, it isn’t a priori obvious the conditions under which cast shadows will help or hinder disambiguation of object properties and relationships. One of the ways of assessing the use and strength of one source of visual information or constraint is to see how one competes with another. In this study, we describe a set of experiments that pit a general lighting principle against a general viewpoint constraint.

Shadows, alongside a myriad of other visual components, compose the images received by the eyes. Images themselves are complicated functions of viewpoint, lighting, surface shape, material and spatial arrangement of the viewed scene. In some sense, vision is the process of inverting this function in various ways for purposes like recognition, classification, grasping, or navigating (Kersten, 1997). Because image formation itself is complex, investigating the reverse process seems to be even more so. One solution has been to isolate a single component of the image formation equation, and understand how that could be inverted. Consider the example of the extraction of 3D shape properties from the projected 2D image. Because of the many-to-one mapping of objects to an image, image information is formally ambiguous about the structure of the objects. This ambiguity can be reduced by incorporating additional constraints or assumptions about the nature of objects and how they project to the image (e.g., Sun & Perona, 1996; Wertheimer, 1923). In terms of their visual experience, humans and/or their visual system have acquired practice in how lighting and viewpoint act together to produce images.

Previous work has shown that the *principle of general viewpoint* provides a useful guide for understanding how the visual system disambiguates the 3D shapes of objects (Lowe, 1987a, 1987b; Nakayama & Shimojo, 1992, see section “General Viewpoint Constraints”). Similarly, considering how illumination is typically experienced can also bring insight into how our visual system might use the *principle of general illumination* to disambiguate ambiguous scenes (Adams et al., 2004; Casati & Cavanagh, 2019; Hershberger, 1970; Mamassian et al., 1998; Sun & Perona, 1996; see section “General Lighting Constraints”).

## General Viewpoint Constraints

The general viewpoint constraint says that a visual interpretation should assume that a 3D object is being viewed from a general (also called generic) rather than an accidental viewpoint. A consequence of this principle is that for many objects, an accidental view of the object means that small changes in viewpoint lead to qualitative changes in the image (Freeman, 1994), for example, from seeing only one side of a box to seeing suddenly two sides (Nakayama & Shimojo, 1992). In this sense, accidental views are special cases of unlikely views—that is scenes presented from an unusual point of view—which in addition manifest themselves as instabilities in the projection.

## General Lighting Constraints

Analogous to an accidental viewpoint, accidental lighting is an unlikely lighting condition (Freeman, 1994). We mention four examples of unusual static illumination conditions (see many examples in Casati & Cavanagh, 2019). (1) When a point light source produces an accidental alignment of shadows and object boundaries (which can impair object recognition, see for example Fig 6.25, p.160 in Casati & Cavanagh, 2019). (2) When a light source is aligned with viewpoint, in that case it will fail to produce visible cast shadows. (3) When only an entirely ambient light is present, in that case there are no cast shadows (this is the case in most studies of surface perception). (4) When the light source is located below the objects (Ramachandran, 1988; Sun & Perona, 1996). A moving light source can also be understood as an accidental or unlikely illumination condition as the visual system expects light sources to be stationary (Kersten et al., 1996; Kersten et al., 1997; Mamassian et al., 1998). In our study, we tested the influence of alignment or non-alignment of light source with viewpoint on object perception, and we do not explore the consequences of general realistic indirect lighting or accidental alignment of shadows and object boundaries.

We will demonstrate that illumination conditions influence how human observers interpret ambiguous scenes and what expectations the visual system has about illumination. We used computer graphic technology to create pictures of test objects placed in 3D scenes. Those scenes were depicted under different illumination conditions. With this paradigm we tested whether vision uses the *principle of general illumination* to determine shape or other properties of the test objects.

## General Methods for Experiments 1 to 4

Animations and static images were prepared using a graphic software (Wavefront's Advanced Visualizer) on a Silicon Graphics workstation. Each 3D scene was rendered in two different illumination conditions that all included an ambient illumination. The virtual camera (or cyclopean eye; Julesz, 1971) was located directly above the center of the object and directed toward its center, thus the scenes were always presented from an accidental viewpoint (an accidental alignment of camera and object). In the cast-shadow condition, the object cast a shadow on the background because of the presence of a directed light source not aligned with the position of the camera (off-center position). The no-shadow condition could be achieved either by modifying the properties of the directed off-center light and the object so that the illuminated object did not cast any shadow or by placing the directed light source at the same angular position as the camera in the scene (an “accidental” illumination) so that the light source caused no visible shadow in the scene. We used an extended light source (it corresponds to a number of light bulbs placed next to each other) that produced soft cast shadows. Note that scene properties could be manipulated so that the background was affected or not by the directed light source, depending on the experiment, whereas the appearance of the object remained identical under both illumination conditions. All scenes were rendered under orthographic projection (similar to viewing the scene with a very large focal length, as with a tele­photo lens). Thus, the size and 2D shape of an object did not change when the position and/or orientation of the object relative to the camera was changing. All participants were naïve as to the purpose of the experiments and were seated in a darkened room. They viewed the test images or sequence of images on a CRT-monitor. Response fields replaced each test image, allowing participants to choose with a mouse click the response field corresponding to their percept. All experiments lasted less than 10 min. All participants gave informed verbal consent.

## Experiments 1a and 1b. Effect of Cast Shadow on Perception of Shapes Depicted in Pictures

These experiments investigated the disambiguating effect of cast shadows on shape perception of objects presented from an accidental viewpoint.

### Participants

Seventeen participants performed Experiments 1a and 1b on different days. They were all members of the institute or friends. No payment was offered.

### Stimuli, Design, and Procedure

**Figure 1.**
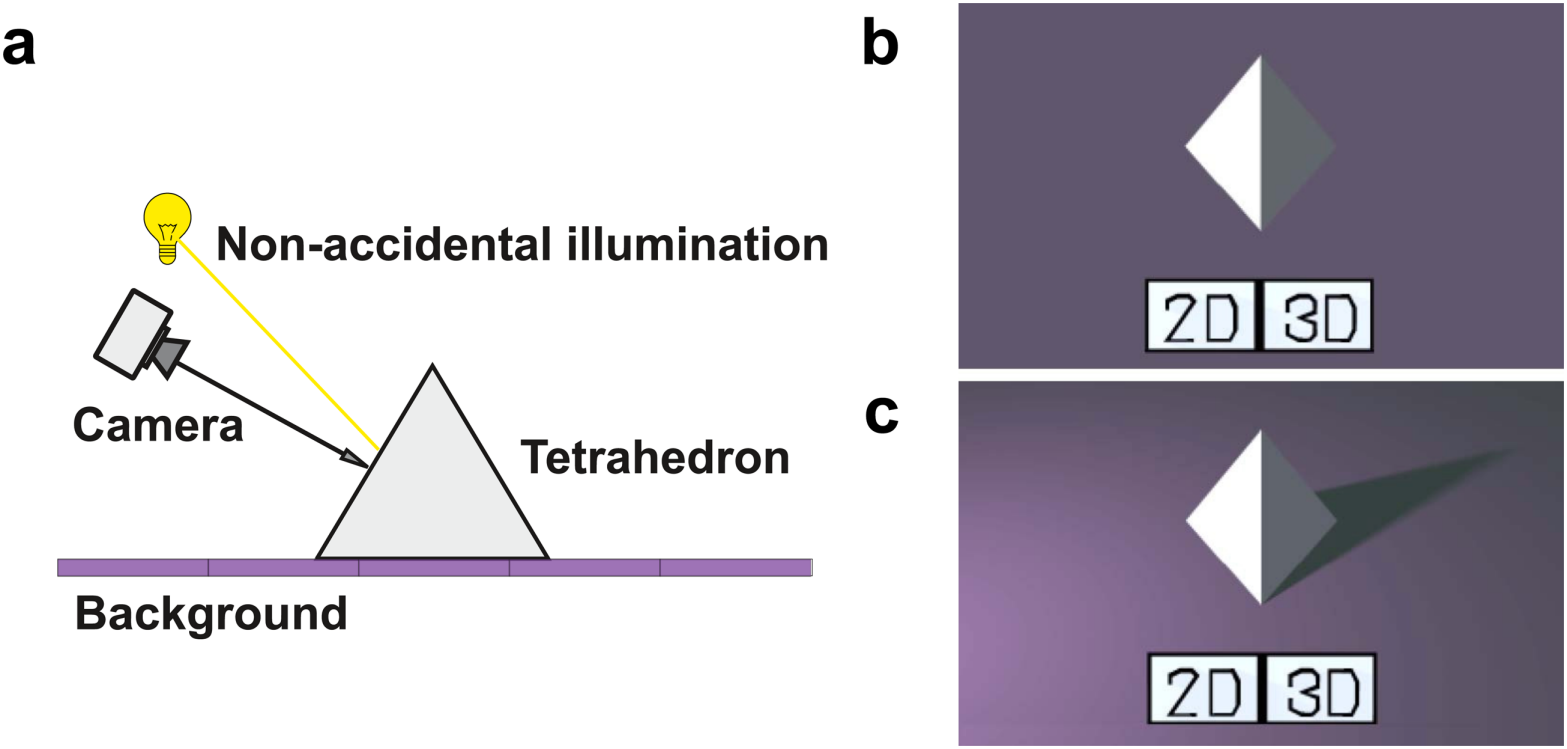
Experiment 1. (a) Side view of the 3D scene used to create the image in (c), the camera is oriented toward the opposite vertex of the hidden face of the tetrahedron, so as to see both visible faces equally. (b) Image in the no-shadow condition, when the light in (a) was aligned with the camera. (c) Image in the cast-shadow condition.

In Experiment 1a, we used two images displaying a uniformly colored tetrahedron placed on a flat background (Figure 1). To create those images, the object was presented under such a viewpoint and illumination that it appears like a diamond with one half dark and one half bright, and an extended light was placed off-center (Figure 1a). In the no-shadow image (Figure 1b), the extended light only illuminated the object, but did not affect the background which displayed no illumination gradient. In that condition, it could be interpreted as a 3D object floating in space while being illuminated from the side or like two painted flat triangles side by side (Sinha & Adelson, 1993). In the cast-shadow image, the light illuminated the object and the background, the volumetric object cast a shadow onto its supporting surface, the background displayed an illumination gradient (Figure 1c). Visual angle was around 10° for each image.

Each participant viewed each stimulus eight times in random order. Each image was presented for 2 s and then replaced by a response image until an answer was entered. Participants’ task was to indicate if the test objects appeared flat (2D), like two mosaic stones set next to each other, or volumetric (3D) by clicking into the corresponding response field. A new trial followed immediately a mouse click. There was no time pressure, but participants were told to answer as correctly and as fast as possible.

To control for illumination gradients in the background, in Experiment 1b, the same 3D scene was used, but we modified the illumination properties to create a third image in which the background displayed the same illumination gradient as in the cast-shadow condition (Figure 1c) but no cast shadow was visible. This new image was shown together with the other two images of Experiment 1a. We created six image sets consisting of the three images displayed side by side, each set with a different placing order of the images. Each set had a visual angle of about 30° wide and 10° high. Each participant viewed each of the six image sets once. In each trial, they listed verbally the three images in order of diminishing volumetric percept. Each stimulus was shown as long as needed. The next trial started after the responses were recorded manually.

### Results and Discussion

#### Experiment 1a

**Figure 2.**
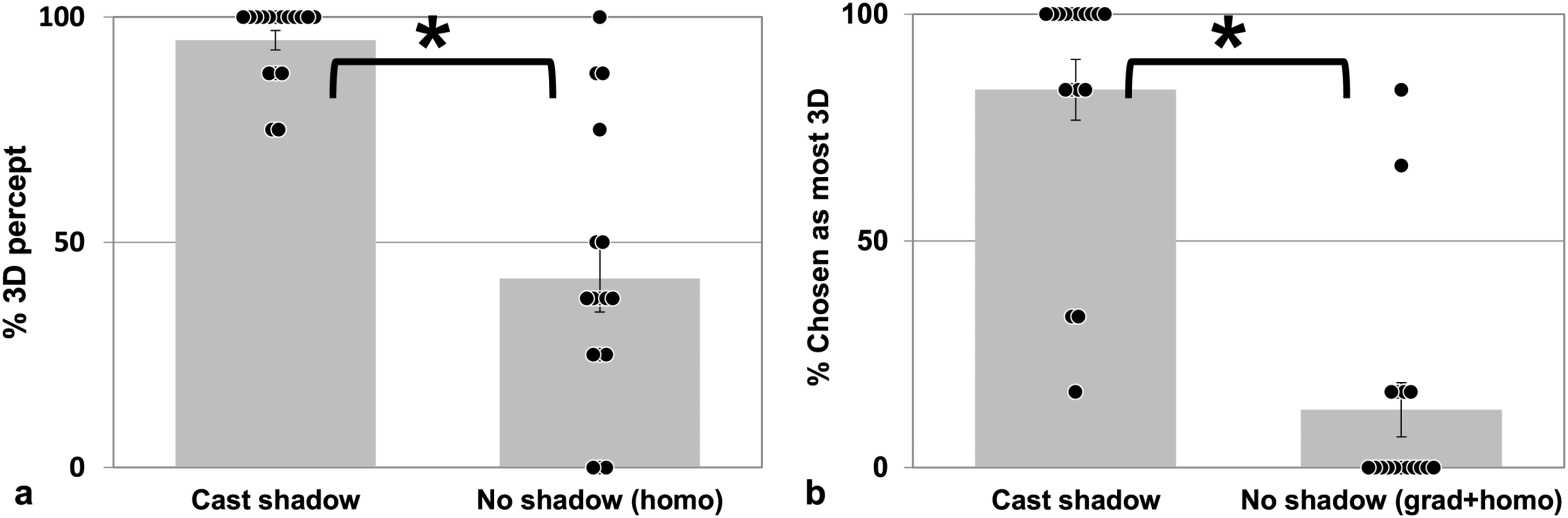
Results of experiment 1. (a) Experiment 1a. Percentage of responses reporting the tetrahedron as 3D (volumetric) when casting a shadow (Cast shadow) or not (No shadow). (b) Experiment 1b: Percentage of responses choosing the tetrahedron with a cast shadow (Cast shadow) or without cast shadow (No shadow) as appearing most volumetric. Black dots depict individual scores, * indicates significance at the 5% level. Grad: background displaying the same illumination gradient as in the cast shadow scene. Homo: homogeneous background with no illumination gradient. Grad: presence of an illumination gradient. Error bars: SEM.

In the no-shadow condition, the observers perceived the tetrahedron either as flat or as 3-dimensional. When a cast shadow was present, the object appeared clearly 3-dimensional (Figure 2a). Paired *t*-tests confirmed that displaying a cast shadow in the scene significantly enhanced the 3D-percept (*t*(16) = 6.628, *p* < .001). This result suggests that shadow information helps crucially volumetric shape perception in 2D pictures. Note that, after the cast shadow cues have led to the abutting triangles being interpreted as being part of one single volumetric object, an observer might entertain this interpretation on later trials even in the absence of the shadow cues. Therefore, our finding may, in fact, be even stronger than what the current data suggest if we would take into consideration only the very first trial of each observer. We did not confine the analysis to those very first trials (in those trials such a bias could not take place) because of the small number of data available. With more data, such an analysis might well have shown a stronger difference between the conditions*.* A possible confound nevertheless might have arisen because the background differed between both test images. We ran Experiment 1b to test whether the effect found depended on this difference.

#### Experiment 1b

To test the importance of the background illumination for the shape percept, we compared how often the tetrahedron was judged to appear as the most volumetric in each of the two no-shadow conditions. The scene displaying an illumination gradient on the background congruent with the shading of the tetrahedron was chosen as depicting a volumetric object in close to 7% of all 102 trials whereas the scene with a homogeneous background was chosen in close to 6% of the trials. A paired *t*-test revealed that this difference was not significant (*t*(16) = 0.156, *p* = .878). It rules out that the background illumination influenced shape percept in our experiment. Importantly, in most trials, participants reported the tetrahedron casting a shadow to appear more volumetric than without it, independently of the appearance of the background (Figure 2b). A paired *t*-test confirmed that the 3D percept was significantly more frequent in the presence of a cast shadow than in the other two conditions pooled together (*t*(16) = 5.825, *p* ≤ .001). These results repeat the finding of Experiment 1a; cast shadows enhance the 3D percept of an object.

## Experiment 2. Effect of Cast Shadow on the Perception of Object Shape in Stereo Pictures

Here we studied the disambiguating effect of cast shadow on the shape perception of objects presented in stereo. We used a “cross” object similar to that used by Nakayama and Shimojo (1992).

### Participants

Seventeen participants performed the experiment. They were all members of the institute or friends. No payment was offered.

### Stimuli

**Figure 3.**
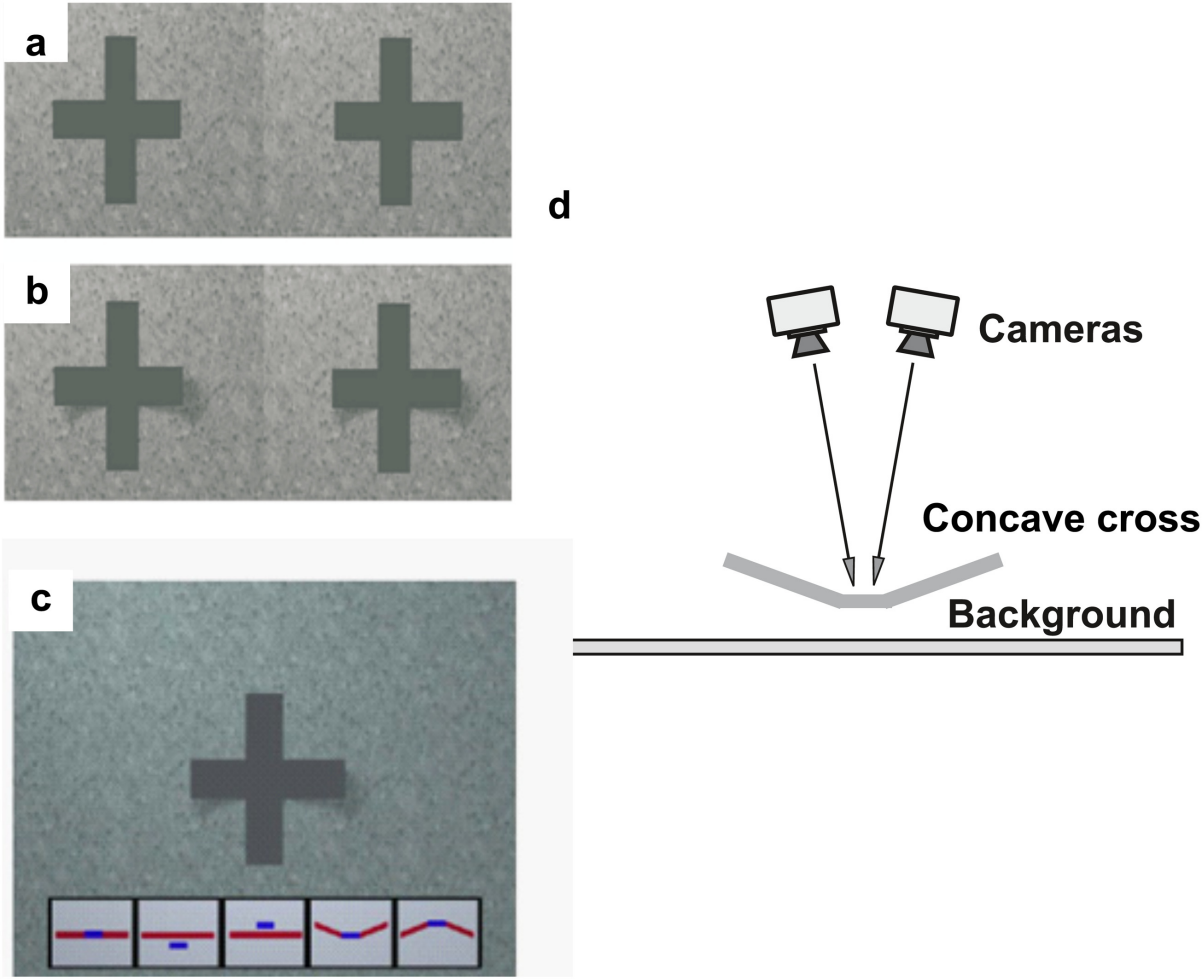
Experiment 2. (a and b) Stereo image pair without/with visible cast shadow. If the image pairs are viewed with crossed disparity, the most common interpretation in the shadow condition is seeing a cross with bent wings. (c) Cross image with icons corresponding to possible perceived shapes of the cross. From left to right: Icon 1—The cross is completely flat, both bars are flat and at the same depth, Icon 2 and 3—The vertical bar is in front of or behind the horizontal bar, Icon 4 and 5—The horizontal wings are bent backward or forward toward the viewer (correct shape percept). (d) Side view of the 3D scene used to create the images.

A 3D “cross” object (Figure 3; Nakayama & Shimojo, 1992) was modeled as two planar oblique-oriented surfaces or “wings” (the horizontal bars in Figure 3a-c) attached to a vertical bar. The whole object surface was of a homogeneous grey color. This cross was lying over a textured grey surface. Two virtual cameras were used to create images for stereo viewing of the scene and an extended light was placed off-center (Figure 3d, extended light not shown). The cameras were placed such that the cross was viewed from an accidental viewpoint by the cyclopean eye of stereoscopic vision (Julesz, 1971). The cross was presented stereoscopically on a CRT-monitor using liquid-crystal shutter glasses (CrystalEyes, StereoGraphics; Hodges, 1992). For the observer, the outer edges of the horizontal limbs had crossed binocular disparity; the horizontal limbs were thus angled inwards, their exterior extremi­ties pointing toward the observer (forming a concave cross), as predicted from linear interpolation of disparity information. Because of the orthographic projection, the size and shape of each bar did not change despite varying distance with respect to the camera. This first shape interpretation is only consistent with the given accidental viewpoint. Another interpretation of the object is that of a horizontal bar extending in front of the vertical bar. This latter interpretation is consistent with the assumption of seeing the cross from a generic viewpoint and is the most frequent one (Nakayama & Shimojo, 1992) in the absence of other shape information.

A stereo image pair of the scene was created for each illumination condition (Figure 3a and b). Visual angle was around 8–10° for each image. Response fields displayed all possible 3D interpretations of the cross shape (Figure 3c). All cross shapes were consistent with the 2D appearance of the object, but only one of them was consistent with the concave 3D shape.

### Design and Procedure

We explained to the participants the various cross shapes depicted in the response fields (see Figure 3c) before the experiment. Thereafter, each stimulus was shown five times to create 10 trials shown in random order. Each trial consisted of a stimulus shown for 4 s and followed by the response image until an answer was entered. Participant clicked into one of the response fields corresponding best to their percept. A new trial started immediately following a response click. There was no time pressure, but participants were told to answer as correctly and as fast as possible.

### Results and Discussion

**Figure 4.**
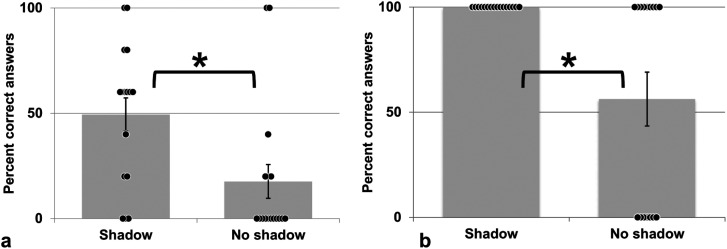
Shape percept of the cross with and without cast shadow. (a) Experiment 2 (static stereo presentation). (b) Experiment 3 (cross in motion, no stereo). Black dots depict individual scores, * indicates significance at the 5% level. Error bars: SEM.

In this paradigm, depth information is available to infer the true shape of the cross object even without cast shadow (Figure 3a and b), whereas relying solely on the 2D shape (the silhouette) could not inform about the true 3D shape. Observers’ responses were categorized as correct when participants clicked on the response field corresponding to the concave cross. All other responses were counted together as wrong. As shown in Figure 4a, the concave cross percept was reported more often when the cross cast a shadow onto the background than in the no shadow condition. A paired*t*-test confirmed that concave shape perception was significantly increased by the presence of cast shadow (*t*(16) = 4.136, *p* < .001). Cast shadow helps disambiguate shape perception and overcome the assumption of a generic viewpoint. The assumption of such a generic viewpoint would lead to perceiving the cross exclusively as a vertical bar behind the horizontal bar (Nakayama & Shimojo, 1992).

In their paper, Nakayama and Shimojo (1992) reported that most observers saw the horizontal limbs lying flat (not bent) in front of the vertical limbs of the cross. Only this percept is consistent with the probable case of seeing the cross from a generic viewpoint. They did not consider the possible role of cast shadows. In our experiment, for trials devoid of shadows, the choices for both percepts (horizontal limb in front: 18%, SEM = 8, concave cross: 26%, SEM = 8) did not differ from each other (*t*(16) = 0.625, *p* = 541). When shadows were present, our participants reported significantly more often the concave percept (49%, SEM = 8) than the percept described by these authors: (17%, SEM = 6; *t*(16) = 2.774. *p* = .014). Note that the concave percept does not conflict with the (sparse and ambiguous) stereo information. Shadow information indicates which of the shape interpretations consistent with the stereo information is correct for a scene interpretation in adequation with both stereo and shadow information. A previous study has shown that inconsistent stereo information can be obliterated when familiar 3D objects are shown, indicating that even early visual processing (stereo processing) can be impacted by higher level processes (object recognition) (Bülthoff et al., 1998). Here, we do not have conflicting information between stereo and shadow cues; our results demonstrate that our visual system is exquisite at using not only stereo information, but all available information, even slight shadows, to choose the correct interpretation of the visual world. This is important to keep in mind as in computational vision, shadows are often removed as they are considered to be cluttering the images.

The high level of inappropriate responses (reporting an impossible percept, e.g., vertical bar in front, flat cross or convex cross) suggests that the observers may not have achieved good stereo fusion. To confirm the importance of cast shadow for shape perception of that cross object, we used the same scene and illumination condition in the next experiment, but replaced stereo presentation by slight motion of the cross.

## Experiment 3. Effect of Cast Shadow on Shape Perception of Moving Objects

**Figure 5.**
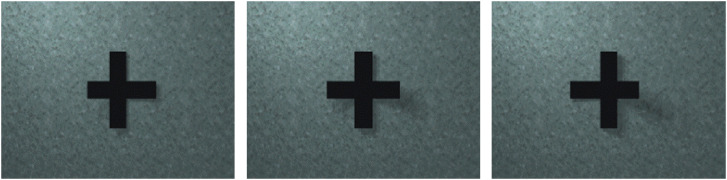
Experiment 3. Some frames of the cast-shadow sequence showing a moving cross. Movie 1 and movie 2 show sequences with and without cast shadow. Clicking on the first image will show Movie 1, clicking on the third image will show Movie 2.

We replaced the stereo presentation by slight oscillations of the cross around its vertical axis in the 3D scene (click on Movie 1 and Movie 2 in Figure 5 to see the videos). In that case, in the absence of cast shadows, the true shape of the cross could be also inferred from structure from motion information.

**Figure:** Movie 1.

**Figure:** Movie 2.

### Participants

Sixteen participants performed Experiment 3. They were all members of the institute or friends. No payment was offered.

### Stimuli

The same scene and illumination conditions as in Experiment 2 were used, except that the background in the shadow condition displayed a visible illumination gradient consistent with the cast shadows. The cross was animated and a single virtual camera pointing toward the center of the object (accidental viewpoint) recorded the motion. We created two videos of 28 frames each lasting each around 1 s. The cross was seen rotating around its vertical axis from left to right and back once (Figure 5) either with or without soft cast shadows. A response image was created as described in Experiment 2. Visual angle was around 10° for each image.

### Design and Procedure

Participants received the same instructions as in Experiment 2 about the task and the meaning of the response fields. Thereafter, each video was shown once, and the order was counterbalanced. Each animation was followed by the response image. There was no time pressure, but participants were told to answer as correctly and as fast as possible.

### Results and Discussion

As is visible in Figure 4b, participants chose the correct shape interpretation of the cross in all trials in the cast shadow condition whereas correct responses were slightly less than 60% in the absence of shadow information. Paired *t*-tests confirmed that shadow information enhanced significantly correct shape percept (*t*(15) = 3.416, *p *= .004). Participants reported verbally that, in the no-shadow condition, the cross often appeared as not rigid, both limbs seemed to slide against each other, whereas their percept was clearly rigid when the cast shadow was shown. Nevertheless, even in the absence of shadow information, structure from motion information allowed them to choose the correct shape significantly more often than by chance (one-sample *t*-test against chance level (20%; *t*(15) = 2.830, *p* = .013). Correct shape perception was significantly improved by the presence of cast shadows. It is noteworthy that we found a strong enhancement of the concave cross percept even though our results show that participants could also extract shape information given solely by structure from motion information.

The response corresponding to the percept described by Nakayama and Shimojo (1992) was reported seldomly and only in the absence of shadow (13%, SEM = 8.5%). We cannot comment on that differing finding, as structure from motion gave strong cues about the veridical shape despite the accidental viewpoint in our experiment.

The results of Experiments 2 and 3 indicate that the general view principle needs to be elaborated to include the principle of general lighting. By itself, the general viewpoint assumption rules out the probability of a concave cross and biases the visual system to interpret the object as a cross made of a horizontal bar being in front and not touching the vertical bar. However, our results demonstrate that this general viewpoint assumption can be overturned by the presence of shadow information.

## Experiment 4. Effect of Cast Shadow on the Perception of Object Rigidity

Here we studied the disambiguating effect of cast shadow on the perception of object rigidity.

### Participants

Twenty-four participants performed Experiment 4. They were all members of the institute or friends. No payment was offered.

### Stimuli

**Figure 6.**
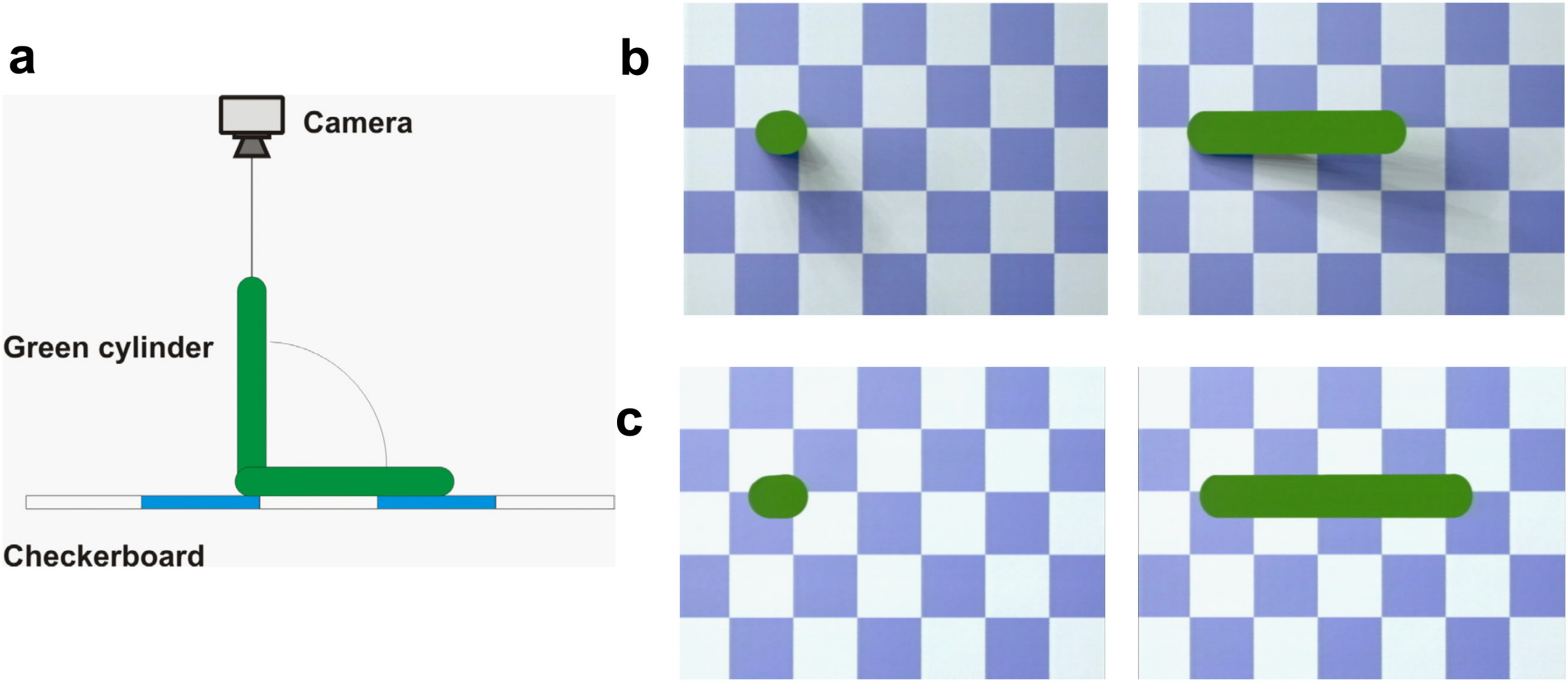
Experiment 4. (a) Side view of the 3D scene used to create the images. (b and c) Two frames of the cast-shadow (b) and no-shadow (c) sequences. Movie 3 and movie 4 show sequences with and without cast shadow.

We created a 3D scene with a flat checkerboard surface on which a cylinder stood, fell and moved back to its starting position. A virtual camera was centered onto the top of the vertical cylinder and aligned with its vertical axis (accidental viewpoint). It recorded the scene to produce two 50-frame animations, one where the moving cylinder casts a soft shadow onto the surface and one without shadows (Click on Movie 3 and Movie 4 in Figure 6 to see a video). In both animations, the background floor did not display any illumination gradient (Figure 6a and c). A “response” image was created depicting the same checkerboard surface with a response field for each possible interpretation of object rigidity (rigid, deforming). Visual angle was around 10× for each image.

**Figure:** Movie 3.

**Figure:** Movie 4.

### Design and Procedure

One trial consisted of the no shadow sequence repeated three times and the other one consisted of the shadow sequence repeated three times. Participants were told that they would see an animated short sequence and that they would be asked about the rigidity of the object in this sequence. The object might appear to deform (change shape) during the sequence or to be rigid and fall. The animation started by pressing a mouse button. Participants clicked into one of the two response field corresponding to their percept. Each trial was shown once. Half of the participants saw the shadow sequence first; the other half saw it last.

### Results and Discussion

**Figure 7.**
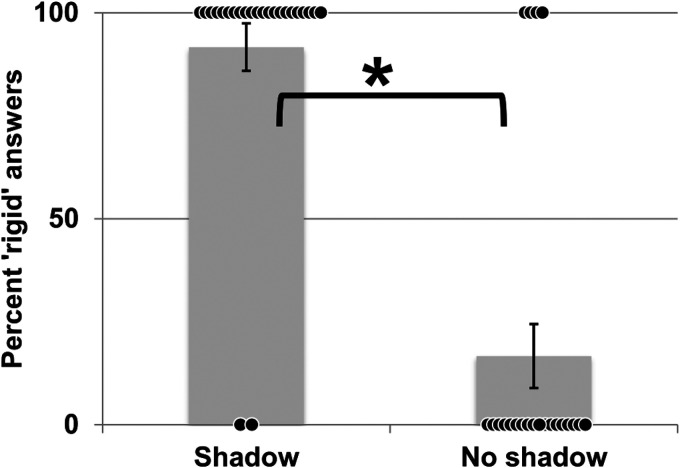
Results of experiment 4. Percentage of responses reporting the green cylinder to be rigid when casting a shadow(Shadow) or not (No shadow). Black dots depict individual scores, * indicates significance at the 5% level. Error bars: SEM.

The results in Figure 7 indicate that most subjects judged the cylinder to be expanding and contracting in the absence of shadow. This percept changed significantly when a cast shadow was present. It was reported to be a rigid object falling onto the floor by more than 90% of the viewers. Again, the percept changed significantly (*t*(23) = 8.307, *p* < .001). The influence of cast shadow was even more dramatic if we look at the responses of 11 participants who saw first the trial without cast shadow. All of them reported seeing a deforming object. In contrast, a third of the 12 other participants who had first experienced the shadow sequence were biased to perceive the object to be rigid also in the following sequence without shadow. In sum, the synchronized visual 2D-deformation of the object with the motion of the shadow strongly bias participants toward the falling rigid cylinder interpretation.

## Experiments 5a and 5b. Effect of Shadows and Shading on Face Perception

So far, we have tested the importance of cast shadows with very simple objects which lacked any shading because of their plane and smooth surfaces. Under such conditions, cast shadows were revealed to be of crucial importance in disambiguating object structure and properties. In this last experiment, we investigated whether the combination of shading and cast shadow information might help to correctly perceive much more complex shapes. We used faces as they are known to be objects of expertise for humans (Sheinberg & Tarr, 2010; Tanaka, 2001). Because faces are socially highly important, humans need to tell apart unfamiliar from familiar faces and to recognize the latter at the individual level. How much can shadow and shading information help to tell apart very similar faces? Here, participants personally familiar with a person had to tell apart that person's face from the same face with a nose strongly deformed along the line of sight. Shadows and shading were indicative of which face had the correct familiar nose, other clues were minimal. The presence of shadows was manipulated by changing the direction of illumination. In Experiment 5a, all faces were displayed with strong cast shadows. In Experiment 5b, we added faces with minimal cast shadows to test shading information alone and also added faces with texture to evaluate the importance of texture information.

### Material and Methods

#### Participants

In Experiment 5a, we tested 22 naïve participants who all were members of the same department. Their participation was voluntary during a weekly lab meeting. In Experiment 5b, we tested 32 naïve participants outside of the department whose participation was voluntary. All participants were personally familiar with the person whose face was used in the experiment. No payment was offered.

#### Stimuli

**Figure 8.**
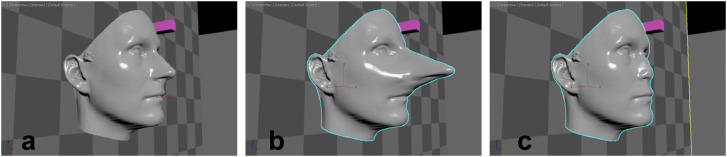
Experiment 5. Profile views of the three faces in the scene. Surface material, illumination conditions, and viewpoints are not those used in the experiment. (a) Original face, (b) Face with long nose, (c) Face with short nose.

The face of one of the authors was used to create the stimuli. It had been 3D-scanned and the 3D shape could be manipulated with an in-house software (morphable model, Blanz & Vetter, 1999; Troje & Bülthoff, 1996). With this software, we generated three versions of that face: the true unchanged face, the same face with an area including the nose deformed to create a strongly elongated nose and the face with an extremely shortened nose (Figure 8). We used extreme deformation to create two faces which could not exist naturally. The purpose of those manipulations was to obtain strong differences in shading and shadows cast by the nose in the test images. In all test images, the nose was presented from an accidental viewpoint reducing nose shape information to a minimum except for shadows and shading. We removed the original texture (surface information) and replaced it by a uniform smooth grey texture in Experiment 5a to focus participants’ attention to 3D shape whereas half the faces had their original texture in Experiment 5b to evaluate the importance of texture information. The magenta blocks provided additional information about light position.

**Figure 9.**
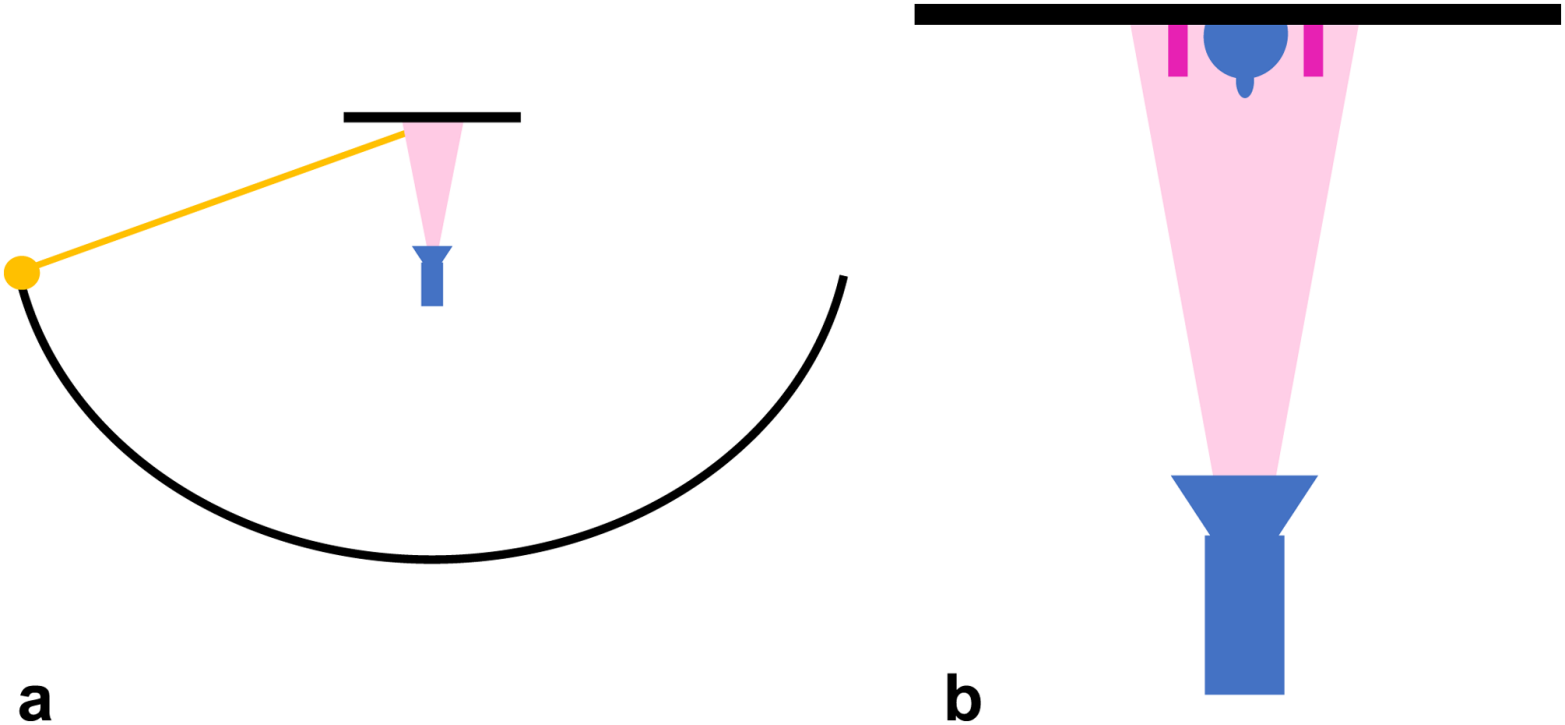
Experiment 5. Scene used to create the test images. (a) Top view showing the background (black rectangle), the virtual camera (blue object) with its field of view (pink), and the semi-arc path (black semi-circle) of the light source (yellow). The face and the magenta blocks are not shown. (b) Close-up view with the face and the flanking blocks.

**Figure 10.**
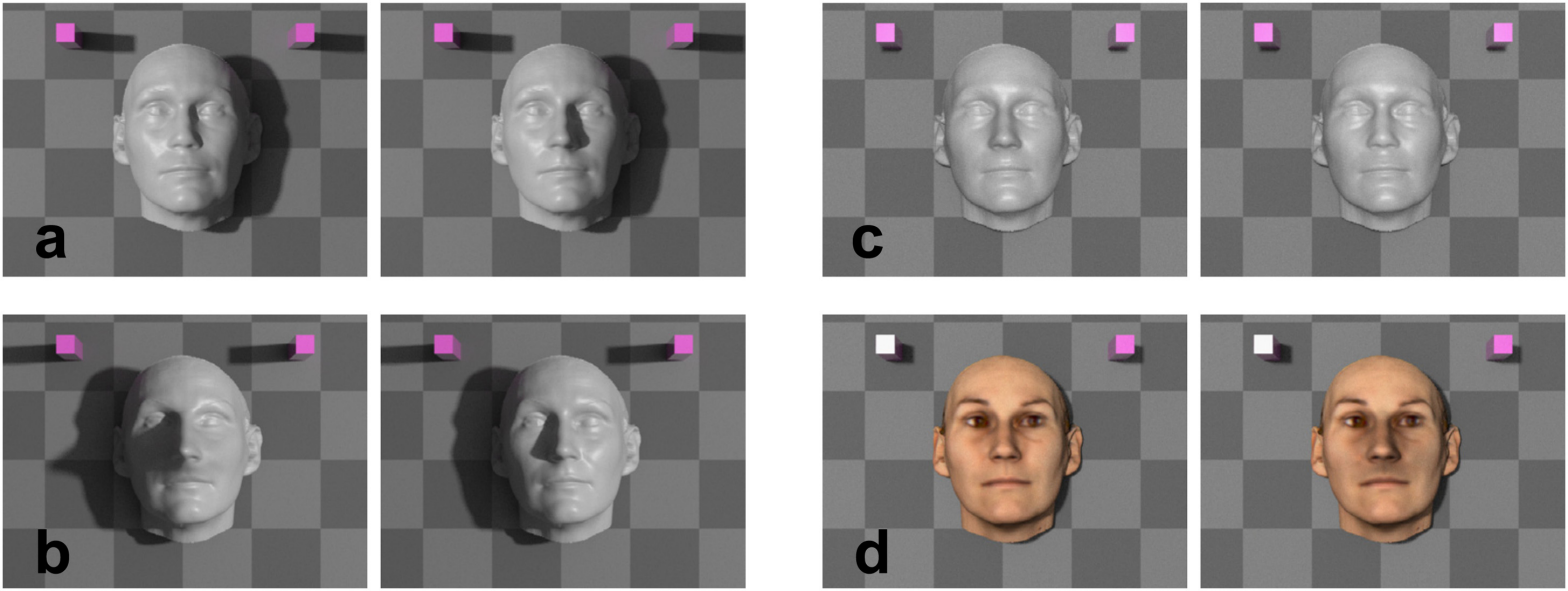
(a and b) Experiment 5a. Example of two trials. Both images of a trial share the same illumination condition; illumination condition differs between (a and b). (a) The shadows cast onto the background in both images display identical outlines (ident cast shad trials). The face on the left is face c in Figure 8 (short nose face), the right face is the original face (face a in Figure 8). (b) Both cast shadows differ strongly from each other (explicit trials). The face on the left is face b (long nose face) in Figure 8, the other face is true face a. (c and d) Experiment 5b. Example of two trials with strongly reduced cast and attached shadow information. Both images in a trial share the same illumination condition, illumination condition differs between (c and d). (c) The face on the right is face c in Figure 8 (short nose face), the other face is the original face (face a in Figure 8). (d) Trial with original facial texture present. The face on the left is true face a in Figure 8, the other face is face b in Figure 8 (long nose face).

We used Autodesk 3ds Max 2018 to create test images. A single face and two magenta blocks were placed in the center of a checkered background (Figures 8–10). The scene was illuminated by two light sources. An area light positioned at various locations created shadows, while an indirect illumination provided a diffuse ambient illumination. The area light was either centered over the nose, which is also the position of the virtual camera or displaced to various positions around the face from (Figure 9a).

For Experiment 5a, we rendered a set of images in which position and orientation of the light was at least 30° away from the line of sight. It induced potentially informative shadows of the nose onto the face and/or onto the background. We use the following terminology: Attached shadows are shadows projected onto the object itself whereas cast shadows depict shadows cast from the object onto the background (Knill et al., 1997; Mamassian et al., 1998). Shading and shadows cast by the nose onto the face or the background gave information about the shape of the nose. Shading information was partially occluded by the cast shadows when the lighting was not aligned with the point of view.

In Experiment 5b, we also rendered images of the scene with the light aligned with or close to (13° away) the viewing axis. In those illumination conditions, the nose cast very little attached shadows onto the face. Slight illumination variations were used to avoid presenting the exact same image multiple times. In those images, shading was the major source of information about face shape.

#### Design and Procedure

Participants were told that they would see two faces shown next to each other in each trial, one face being the true face and the other a manipulated version of that face. Their task was to determine which face was the true face. The faces in each trial were arranged left and right of the center of the screen. Left/right position of the original face images was approximately balanced. In each experiment, the pseudo-random order of the trials was the same for all participants. No feed-back was given. Each trial lasted for 5 s, followed by a blank screen for 3 s during which participants entered their answer on a response sheet or gave their response orally. Presentation was done with PowerPoint with automatic timing. In Experiment 5a, one training trial was followed by 52 test trials. The test images were shown on a projection screen. Participants were seated at various distances (2 to 5 m) from the projection screen. In Experiment 5b, two training trials were followed by 33 test trials and participants performed that experiment on a computer monitor.

In Experiment 5a, each trial displayed faces differing from each other in their shading and attached shadows. Importantly, in one type of trials (*same cast shad* trials), the nose never cast a shadow onto the background, therefore both faces projected identical cast shadows onto the background (Figure 10a). In the other trials (*explicit* trials) that always presented a long nose face as a distracter, the cast shadows of both faces differed strongly (Figure 10b). The nose of the long nose face projected a visible cast shadow which revealed its unnatural length. Those *explicit* trials were expected to facilitate correct responses. In our stimuli, original and short nose faces never projected nose shadows onto the background. The experiment lasted about 15 min.

In Experiment 5b, test faces were with (Figure 10d) or without facial texture (Figure 10a and c). In 2/3 of the trials, cast shadows were reduced to a minimum (*no cast shad*), the light was either exactly aligned with the camera (Figure 10c), or slightly displaced by *13 degrees away from the line of sight,* which created very small attached cast shadows (Figure 10d). These slight illumination changes were used to avoid repeating exactly the same stimuli many times. The remaining 1/3 of the trials were *same cast shad* trials as those used in Experiment 5a (Figure 10a). We added faces displaying texture information as stimuli for three reasons. First, textured faces look more natural and their processing might differ from non-textured faces, resulting in potentially differing usefulness of shading for the task. Second, we wanted to test textured faces as the authors of a study using textured faces report finding no evidence that shading cues helped to deduce nose shape (Schumacher & Blanz, 2012). Last, adding texture to some of the faces reduced the monotony of showing very similar images during the test. The experiment lasted about 10 min.

### Results of Experiment 5a

On average, 68% of the answers were correct (participants’ range: 46–85%, SEM 2.03, STDV 9.50). This is significantly better than chance level (50%, one-sample *t*-test: *t*(21) = 9,024, *p* ≤ .001).

**Figure 11.**
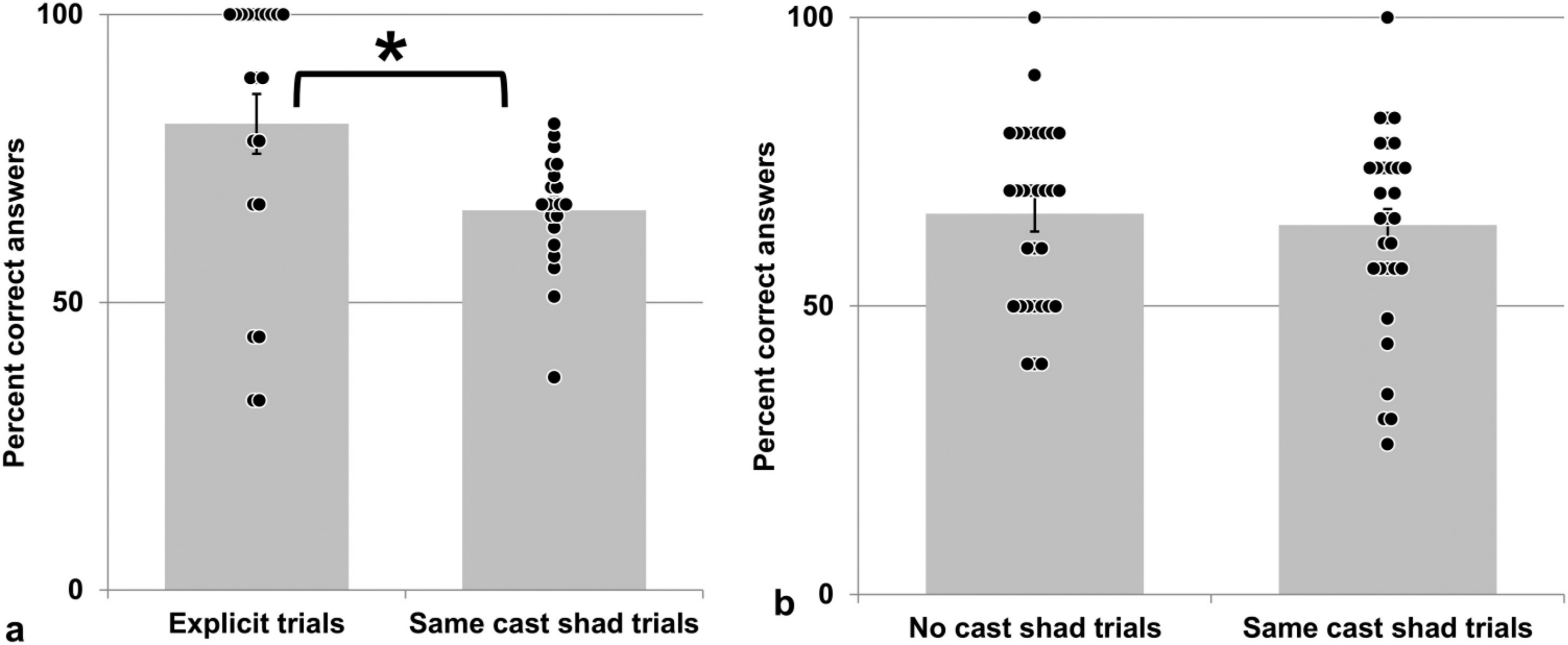
(a) Results of experiment 5a. (b) Results of experiment 5b. Error bars: SEM, *: paired *t*-test with *p* < .05. Black dots depict individual scores, * indicates significance at the 5% level. For more details, see text.

One participant did not follow the instructions, we removed all his data from further analyses. Average accuracy of the remaining 21 participants was better than chance level for each trial type (one-sample *t*-tests: *t*s(20) ≥ 6.032, *p*s ≤ .001). Mean performance for *explicit* trials (displaying faces with differing cast shadows) was significantly better than for trials with identical cast shadows (Figure 11a, paired *t*-test: *t*(20) = 2.706, *p* = .014). Informal debriefing revealed that no participant was aware in which way both faces differed or reported using shadows for performing the task.

### Results of Experiment 5b

Across participants, 64% of the answers were correct (participants’ range: 33–94%, SEM 2.06, STDV 9.50). This is significantly better than chance level (50%, one-sample *t*-test: *t*(30) = 5,446, *p* ≤ .001).

Adding texture to the face stimuli in *No cast shad* trials did not influence significantly participants’ responses (no texture: 67% correct, texture present: 60%, paired *t*-test: *t*(30) = 1.949, *p* = .061). Therefore, we analyzed these two types of stimuli together. Participants responded equally well in trials with (66%) and without large attached shadows (64%, paired *t*-test: *t*(30) = .613, *p* = .544, Figure 11b). Note that there were no *explicit* trials in this experiment. Debriefing revealed that none of the participants reported using shadows knowingly to perform the task. No participant (except one) reported that both noses differed in some way.

### Discussion

We were interested in the usability of shadows and shading for inferring face 3D shape in the absence of facial texture. Participants saw side by side a familiar face shape and the same face with a deformed nose. They had to indicate which one was the true face of their colleague. Because of the accidental viewpoint, information about nose shape differences between both faces was given mainly by the shading and the cast shadows.

In Experiment 5a, participants could indeed use and interpret correctly shading and attached shadow differences although they were not aware in which way both faces differed and that shading and shadows were the main indicators to use. Our results demonstrate that we are expert at interpreting shadows even without explicitly noticing their informative content for retrieving face shape and that we have a quite precise representation of how shading and shadows should look like in familiar faces.

We also found that participants responded better to explicit trials. In these trials, the shadow contour onto the background gave the viewer explicit additional information about nose shape. Cast shadows can be described as the contour of the face “viewed” by the light source which has a different viewpoint onto the face than the camera (Casati, 2004). Participants used rather automatically this additional information which disambiguates nose shape to achieve better performance for those trials. In contrast, the studies of Mamassian (2004) and Ostrovsky et al. (2005) report that participants do not easily notice inconsistent cast shadows, even when those are brought to attention. In a previous study of Schumacher and Blanz (Schumacher & Blanz, 2012), participants first saw a frontal view of a face and thereafter profile views of two identical faces except that one had a slightly modified nose shape. All images were devoid of cast shadows. Participants chose significantly more often the face with the correct nose over a random nose. In their study, Schumacher and Blanz (2012) also report finding no evidence that their participants used shading information for their task. Our experiment 5a adds that participants can use shadows and shading to choose the correct face in the absence of texture information.

We tested faces with shading but with no or minimal attached shadows in Experiment 5b. Our results demonstrate that participants could use shading information alone to choose the correct face and that the presence of face texture did not help significantly in comparison to trials lacking that information. As we used much stronger, more unnatural nose deformation than used by Schumacher and Blanz in their study (2012), all trials in Experiments 5a and 5b displayed shading and cast shadows that differed a lot between both test faces. More subtle facial deformations might not be noticed with shading information only. Because of differences between the study of Schumacher and Blanz (2012) and ours, the question about the role of facial texture in assessing facial shape remains open.

## General Discussion

In all experiments, participants saw accidental views of objects presented in static or dynamic scenes. When simple geometric objects (Experiments 1–4) did not cast shadows, visual information was insufficient for the observer to make an informed choice about properties of the test object and they gave a variety of shape interpretations. When shadow information was available in the same scenes, the majority of the observers were able to use this disambiguating information and they chose in majority the shape interpretation compatible with the shadow information. In the presence of cast shadows, a tetrahedron was more likely to be perceived as a volumetric object than a painted surface, the shape of a concave cross seen in stereo or in motion was more likely to be perceived correctly and a falling cylinder was more likely to be perceived as a solid object. Note that in our stimuli, the match between object and cast shadow was always evident and the shadows were not manipulated in terms of, for example, their blurriness or their lightness/darkness (see the recent study of Cavanagh et al., 2021, for their roles in depth perception). When more complex objects (faces, Experiment 5) were used, shading information about the shape of the face was also present in addition to cast shadows. Participants could use cast shadows and/or shading to choose the correct one of two faces when the distractor face had a strongly deformed nose.

Finally, in view of the strong effect that shadows have on how objects are perceived, two notable facts emerge. (1) How little those shadows or the differences between scene illuminations were noticed by our observers. Most were unaware about what influenced their responses. Unfortunately, we did not register their responses in a systematic fashion, therefore we cannot advance a strong statement about the “non-noticeability” of those potent cues. (2) In the first four experiments, we used soft shadows that are quite fuzzy with only gradual luminance changes in the images. In our experiments, those soft shadows do not give a clear additional view of the object. What is crucial in terms of their property for them to be so powerful cues in our study? We would argue that noticeability, at least, is not necessary. In some cases, it might even lessen their strength as cue (Kersten et al., 1996). Numerous studies have reported that we are not very good at detecting when a shadow is inconsistent in an image (Casati & Cavanagh, 2019; Mamassian, 2004; Ostrovsky et al., 2005). What makes a shadow ineffective or less potent? Even white shadows or other objects in a scene can act as a shadow under certain circumstances (Kersten et al., 1997). These considerations suggest that there is more to shadows than offering another clear noticeable view of an object to detect its properties like solidity or shape.

In sum, our results support the notion that image interpretation does not occur purely bottom-up. When explicit information is missing for simple objects, various interpretations of a given scene occur. When additional shadow information is given, scene understanding can be summarized as finding one solution compatible with prior knowledge about general lighting (Kersten et al., 2004). For more complex objects (faces), participants used successfully their knowledge about how light illuminates familiar faces to choose which of two faces was the correct face even though they were not aware of differing shadows. We conclude that general lighting reduces ambiguity in accidental views.
